# Comparative assessment of solvents toxicity using early life stages of amphibians and cell lines: a case study with dimethyl sulfoxide

**DOI:** 10.3389/ftox.2025.1672301

**Published:** 2026-01-05

**Authors:** Sónia Dias Coelho, Diana Campos, Mónica Almeida, Carla Quintaneiro, Miguel Oliveira, Isabel Lopes

**Affiliations:** Centre for Environmental and Marine Studies (CESAM) and Department of Biology, University of Aveiro, Aveiro, Portugal

**Keywords:** non-animal alternatives, anura, hazard assessment, *In vitro* methods, amphibian ecotoxicology

## Abstract

The reduction in the number of animals being used in experimental assays has been a concern of the scientific community. In this sense, non-animal alternative methods have been increasingly tested. This study intended to explore how cell-based responses compare to organismal outcomes and if the former models could contribute to minimizing the number of live animals needed in subsequent stages of hazard/risk assessment of chemicals on amphibians. For this, the toxicity of the commonly used solvent dimethyl sulfoxide (DMSO) was assessed in early life stages (embryos and tadpoles) of two anuran species (*Xenopus laevis* and *Pelophylax perezi*) and in 2 cell lines of *X. laevis* (A6 and XTC-2). In the *in vivo* assays, mortality, teratogenic effects, and biometric parameters were evaluated, while for *in vitro* assays, the assessed endpoint was viability. Overall, the obtained data suggest similar sensitivity of both species and life stages to DMSO. The 96 h-LC_50_ estimated for embryos and tadpoles were, respectively, 2.19% and 2.56% for *X. laevis* and 3.19 and 3.41 for *P. perezi*. The solvent DMSO induced several malformations in early life stages, which may have implications for the fitness of organisms at later stages. A slightly higher sensitivity to DMSO was observed in the *in vivo* approaches comparatively to *in vitro* approach (72 h-LC_50_ of 3.10% and 2.62% for A6 and XTC-2, respectively), though it can not be considered significantly different. As such, it is suggested that the latter approach may be considered to serve for first screenings of the ecotoxicity of organic solvents. Such a strategy of using *in vitro* assays as screening tools, has the potential to reduce the number of animals to be used in subsequent *in vivo* testing phases by providing information for the refinement of concentrations to be tested in *in vivo* assays, thereby supporting both reduction and replacement objectives.

## 1 Introduction

Anthropogenic activities have long been recognized as detrimental to ecosystems and human health, with many substances developed to improve human wellbeing showing toxic potential to biota. In 2023, the European Union (EU) produced 68 million tonnes of chemicals considered as hazardous to the environment, with consumption reaching 57.3 million tonnes (Eurostat, 2024). Though these numbers were the lowest since 2004, they remain concerning due to limited knowledge about the ecological impacts of numerous chemicals and the constant emergence of new contaminants, underscoring the need for robust hazard assessment strategies. Numerous guidelines have been established to assess the toxicity of chemicals; many of these include animal experimentation involving vertebrate models such as amphibians (e.g., ASTM, 2012; ASTM, 2014; OECD, 2009). Ethical concerns surrounding such practices have fostered international efforts to develop and validate non-animal alternative methods, including *in vitro*, *in silico*, and *in chemico* approaches (Kandarova, 2021). These initiatives align with the 3R’s principles (Replace, Reduce, and Refine) to promote more humane and sustainable testing practices (Lauwereyns et al., 2024). In line with this, the EU REACH regulation favours the use of alternative methods over the conventional *in vivo* assays, if validated or in pre-validation status and developed under standard conditions (Lilienblum et al., 2008). *In vitro* assays, recognized by EFSA (European Food Safety Authority) as good “new approach methodologies” (Miccoli et al., 2022), have been successfully applied to several groups of vertebrates, namely, fish and mammals. These methods not only address ethical concerns but also offer greater cost-effectiveness, faster testing times, and improved reproducibility compared to traditional animal testing approaches. *In vitro* cell culture systems require lower resource investments as it regards costs for animal procurement, housing, veterinary care, and specialized facility maintenance. These methods also offer accelerated testing protocols, with results obtainable within days to weeks compared to months required for *in vivo* studies. Furthermore, the standardized and controlled conditions of cell culture systems provide enhanced reproducibility and reduced variability compared to animal models, potentially improving the reliability of toxicological assessments while minimizing the number of tests required. The Organisation for Economic Co-operation and Development (OECD) already published guidelines for testing chemicals using fish cell lines (OECD, 2021); similar frameworks are needed for other vertebrates, particularly amphibians given their vulnerability to environmental pollutants (IUCN, 2023; Martin et al., 2024). Amphibians are among the most threatened vertebrate groups, with around 100 species lost in the past 5 decades and 41% of remaining species at risk of extinction (IPBES, 2019; IUCN, 2023). Understanding the impacts of pollutants on this taxon is therefore critical for their conservation. However, meeting the extensive data demands for risk assessment through traditional animal testing is unsustainable due to ethical and practical constraints. Developing alternative methods that minimize the use of animals from (laboratory and wild populations) is thus essential for advancing amphibian ecotoxicology in a sustainable and ethically responsible manner.

In this context, the present work aimed to assess how cell-based responses compare to organismal outcomes and if the former models could contribute to minimize the number of live animals needed in subsequent stages of hazard/risk assessment of chemicals on amphibians.

Within the aforementioned guidelines, the organic solvent dimethyl sulfoxide (DMSO) is a model chemical commonly used to solubilize numerous hydrophobic compounds, for both *in vitro* and *in vivo* studies, due to its amphipathic nature (Verheijen et al., 2019). It also serves as a cryoprotectant, preventing membrane damage during cell freezing (Niebergall-Roth and Kluth, 2025). Given these characteristics and its assumed low toxicity (<10% v/v), it is commonly used in toxicological and pharmacological studies (Galvao et al., 2014; Verheijen et al., 2019). The OCDE recommends using a solvent concentration not exceeding 0.01% v/v (OECD, 2019). However, higher levels are often required to solubilize highly hydrophobic compounds and are not always reported (Galvao et al., 2014; Hoyberghs et al., 2021). Considering this framework, DMSO was selected as a model chemical to attain the main goal of this study and as well as to provide evidence for its recommended concentrations regarding ecotoxicology studies with highly hydrophobic compounds.

In this context, the present study aimed at: (i) comparatively assess the toxicity of DMSO using *in vivo* and *in vitro* amphibian biological models and (ii) evaluate the potential of *in vitro* assays, with cell lines of *X. laevis*, as suitable non-animal alternatives to firstly screening chemicals toxicity, and thereby contribute to reduce the needed number of *in vivo* assays, with early life stages of amphibians, at subsequent stages of the risk assessment process. To accomplish these objectives, the toxicity of DMSO was assessed through *in vivo* assays, conducted accordingly with the 3 R’s principle, by using embryos and tadpoles of two anuran species (*X. laevis* and *Pelophylax perezi*), which are non-independent feeding developmental stages, and thus, are considered as non-animal new approach alternatives. Complementary *in vitro* studies were performed using 2 cell lines from *Xenopus laevis* (A6, comprising kidney cells, and XTC-2, comprising fibroblast cells).

## 2 Materials and methods

### 2.1 *In vivo* biological models

The selected anuran species were the clawed frog *X. laevis* (native from sub-tropical/tropical regions), used as a model due to its well described characteristics and development (Nieuwkoop and Faber, 1994), it is a standard species recommended by several OECD guidelines for conducting toxicity tests; and the autochthonous species *P. perezi* (native from temperate regions), the most common and widely spread amphibian species in the Iberian Peninsula, with an IUCN conservation status of Least Concern (IUCN, 2023). The comparison of the sensitivity of lab- and field-originated species to chemicals is of much relevance to validate possible non-animal alternative assays.

*Xenopus laevis* (standard species) embryos and tadpoles were obtained by inducing reproduction of adults from a culture maintained at the Department of Biology facilities from the University of Aveiro, Portugal. Male and female were injected with 100 IU and 500 IU of human chorionic gonadotropin (hCG; CG5-1VL, Pcode:1003093508, Sigma-Aldrich®, Germany), respectively. After about 15 h of amplexus, the viable eggs were selected under a stereomicroscope and transferred to FETAX medium (Dawson and Bantle, 1987). Part of the viable eggs (Nieuwkoop and Faber (NF) stage 8–11 (Nieuwkoop and Faber, 1994)) were immediately used for a toxicity test adapted from the Frog Embryo Teratogenesis Assay–FETAX (ASTM, 2012). The remaining eggs were kept in FETAX medium, at 23 °C ± 1 °C, a photoperiod of 14:10 h light:dark, under continuous aeration and fed with macerated TetraMin® flakes (from the moment they began feeding, stage NF 45) until reaching the stage NF 46. At this stage, they were immediately used to perform the tadpole toxicity assay (adapted from ASTM, 2014).

In the assays performed with *P. perezi* (native species), embryos and tadpoles were obtained from different egg masses collected from a freshwater pond at Quinta da Boavista, Aveiro, Portugal (40°35′48.8″N 8°41′43.4″W) and transported to the laboratory in water from the sampling site (Santos et al., 2013). Once in the laboratory, the egg masses were transferred to FETAX medium, and the viable eggs were selected under a stereomicroscope. Similarly to the approach used for *X. laevis*, a portion of the eggs was immediately used to run the exposure test with embryos in Gosner (G) stages 8–10 (Gosner, 1960) following the FETAX guideline (ASTM, 2012). The remaining viable eggs were kept in FETAX medium (23 °C ± 1 °C, 14:10 h light:dark photoperiod and continuous aeration, fed with macerated TetraMin® flakes) until reaching the stage G 25, to run the tadpole exposure assay based on the ASTM international guideline (ASTM, 2014).

For each species, the DMSO concentration range was determined through preliminary assays to define an interval suitable for the species’ sensitivity and capable of revealing adverse effects and calculating median lethal concentrations in exposed organisms.

#### 2.1.1 Embryo teratogenicity assays

*Xenopus laevis* embryos (at stage NF 8–11) were exposed to the following concentrations of DMSO (CAS: 67–68–5; purity ≥99.7%; Thermo Fisher Scientific® (UK)): 0.89, 1.21, 1.63, 2.20, 2.97, and 4.01% v/v, plus a negative control (FETAX medium only). The test solutions were prepared by diluting a stock solution of DMSO (7%) in FETAX medium. Three replicates were carried out per tested concentration of DMSO and five replicates for the control. Each replicate consisted of 20 embryos (keeping the jelly coat to preserve a more realistic exposure and thus contribute to a more accurate assessment of the hazards posed by the chemicals) distributed in 55 mm Ø Petri dishes, containing 10 mL of test solution. The assay lasted 96 h and was performed at 23 °C ± 1 °C and a 14:10 h light:dark photoperiod with medium renewal after 48 h exposure. Conductivity and pH were measured, with a multiparameter probe (Multi 3410 SET C 2FD45C, Wissenschaftlich Technische Werstätten, Weilheim, Germany), at the beginning and end of the assay, to control the quality of the medium. The assay was monitored every 24 h to check for organisms hatching and mortality. Dead organisms were removed to prevent the growth of microorganisms, preserving the medium quality. After the 96 h of exposure the following endpoints were measured: mortality, hatching, and malformations (observed under a stereomicroscope). Photographs of all surviving organisms were taken to determine body lengths [total body length (TBL) and the snout-to-vent length (SVL)] using ImageJ software (NIH, USA). The tail length (TAL) was estimated by subtracting the SVL from the TBL.

*Pelophylax perezi* embryos, in the G 8–10 stages, were also exposed to six concentrations of DMSO (1.63, 2.20, 2.97, 4.01, 5.41, 7.31% v/v), plus a negative control (FETAX medium only). Solutions were prepared by diluting a stock solution (15%) in FETAX medium. The test duration, procedures and conditions were the same as described for *X. laevis*. After 96 h of exposure, the same endpoints were measured: mortality, hatching, malformations and body lengths (TBL, SVL and TAL), as described for *X. laevis*.

#### 2.1.2 Acute toxicity assay with tadpoles

*Xenopus laevis* tadpoles (at stage NF 46) were exposed to a negative control (FETAX medium) and seven concentrations of DMSO (1.40, 1.68, 2.02, 2.42, 2.90, 3.48, 4.18% v/v), obtained through the dilution of a stock solution (19%) in FETAX medium. Each treatment consisted of five replicates with three tadpoles per replicate. The three organisms were placed in the test vessels filled with 150 mL of test solution. Tadpoles were exposed for 96 h, at 23 °C ± 1 °C and a 14:10 h light:dark photoperiod under continuous aeration. At the beginning of the assay, 15 randomly selected tadpoles were weighed (analytical balance) and measured (as described for embryos) to obtain the initial average values of the pool of tadpoles used for the assay to allow the determination of daily growth increments. Tadpoles were fed with macerated TetraMin® flakes (±2% of the average tadpole body weight), at the beginning of the assay and after 48 h of exposure, when the test solutions were renewed. This procedure was implemented because, by late stage 47, the mouth opening is anatomically functional and tadpoles may begin to ingest food particles. However, the functional capacity of the intestine for absorbing ingested material at these stages is still uncertain (McKeown et al., 2017). Thus, while ingestion may represent a potential additional exposure route for chemicals, its quantitative contribution to systemic uptake remains unclear at this developmental stage. Recognizing this uncertainty is important to ensure that exposure scenarios in ecotoxicity testing realistically reflect the physiological context of early tadpole development. Conductivity and pH were measured, with a multiparameter probe, in the beginning and end of the assay, to control the stability of the medium. Mortality was checked every 24 h and dead organisms were removed. After 96 h, the following endpoints were measured: mortality, malformations, weight, body lengths (TBL, SVL and TAL) and the daily body mass and TBL increment were determined. Daily body mass and TBL increment were determined according to Equations 1, 2, respectively, based on specific growth rate equations (Hopkins, 1992):

$$Daily\;\;body\;\;mass\;\;increment\;\left(mg/day\right)=\frac{\ln Wf-\ln\bar{W}i}{T}$$ (1)

where, *Wf* is the final weight of the tadpoles (mg), $\bar{W}$ *i* is the average of the initial weight of the tadpoles (mg), and *T* is the exposure period (days).

$$Daily\;\;total\;\;body\;\;length\;\;increment\;\left(cm/day\right)=\frac{\ln Lf-\ln\;\bar{L}i}{T},$$ (2)

where, *Lf* is the final TBL of the tadpoles (cm), $\bar{L}$ *i* is the average of the initial TBL of the tadpoles (cm), and *T* is the exposure period (days).

Similarly, *P. perezi* tadpoles at stage G 25 were exposed to a negative control (FETAX medium) and seven concentrations of DMSO (1.63, 1.96, 2.35, 2.82, 3.38, 4.06, 4.87% v/v). The test solutions were prepared by diluting a stock solution (11%) in FETAX medium. The assay duration, procedures, and conditions were the same as for *X. laevis*. After 96 h of exposure, the same endpoints were measured: mortality, malformations, weight, body lengths (TBL, SVL, and TAL), and daily body mass and TBL increments.

At the end of each assay with embryos and tadpoles, all organisms of both species were euthanized with a tricaine methanesulfonate (MS-222; Acros Organics, Thermo Fisher Scientific, UK) overdose.

All *in vivo* experiments were carried out in compliance with the ARRIVE guidelines and the Directive 63/2010 and were authorized by the Portuguese institution responsible for the authorization of animal experimentation, i.e., Direcção Geral de Veterinária e Alimentação (Permit No. 0421/000/000/2022).

### 2.2 *In vitro* models

The amphibian cell lines used in this study were *X. laevis* A6 kidney cells (American Type Culture Collection CCL-102) and *X. laevis* XTC-2 fibroblast cells, both kindly offered by Professor Peter Lorenz (University of Rostock, Germany). Cells were cultured in T75 cell culture flasks and maintained in an incubator at 25 °C, under atmospheric air, using 55% Leibovitz-15 (L-15; Biowest, France) and 35% of sterile ultrapure water (UPW) in the case of A6 cells, and 65% L-15% and 25% sterile UPW for XTC-2 cells (Born et al., 2014). Both media were supplemented with 10% of Fetal Bovine Serum (FBS; Capricorn Scientific, Germany), 100 μg/mL of Streptomycin and 100 μg/mL of Penicillin G (Capricorn Scientific, Germany) and 2.5 μg/mL of Amphotericin B (Gibco, UK). The phosphate-buffered saline (PBS; prepared with chemicals obtained from Sigma-Aldrich®, Germany) used to wash the cells was adjusted to amphibian osmolarity (PBS 70%), according to Bui-Marinos et al. (2020). All experiments were performed between passages 35 and 50.

#### 2.2.1 Cytotoxicity assays with A6 and XTC-2 cell lines of *Xenopus laevis*

To assess the effects of DMSO on cell viability, A6 and XTC-2 cells were plated in 96-well culture plates, at a density of 2.5 × 10^4^ and 1.0 × 10^4^ cells per well, respectively, and allowed to adhere overnight. These cell densities were based on preliminary assays that assessed cell growth rate and optimal absorbance readings. Cells were exposed to ten concentrations of DMSO (0.0625, 0.125, 0.25, 0.5, 1, 2, 4, 6, 8% and 10% v/v) dissolved in the cell culture media and incubated for three different time periods (24, 48 and 72 h). Cellular viability was assessed by the thiazolyl blue tetrazolium bromide (MTT) viability assay adapted from the National Institute of Health (NIH) guide (Ris et al., 2016). Briefly, the adhered cells were exposed to 100 µL of each medium, containing the desired DMSO concentration, at 25 °C. At each time-point, the test solutions were removed, and cells were washed with 70% PBS. Then 100 µL of 3-(4,5-dimethylthiazol-2-yl)-2,5-diphenyltetrazolium bromide (MTT, CAS 298–93–1, purity >98,0%, TCI® Europe, Belgium), at a concentration of 0.5 mg/mL (in 70% PBS, pH 7.4) was added, and cells were incubated for 2 h. Afterwards, the MTT solution was removed and formazan crystals formed inside the cells were solubilized by 100% DMSO (60 µL). Samples were analysed in a microplate reader MultiSkan Spectrum (Thermo Fisher Scientific, USA) at 570 nm using 690 nm as a baseline. Viability was expressed as a percentage of the control. The cytotoxicity was evaluated in three independent experiments, in quadruplicate, for all concentrations.

### 2.3 Data analysis

The median lethal concentrations of DMSO (96 h-LC_50_) for embryos and tadpoles (of both species) were estimated through a three-parameter nonlinear regression model using the SigmaPlot (v14.0) software. When possible, the 96 h median effective concentration (96 h-EC_50_) for malformations, observed in the *in vivo* assays, was determined using the PriProbit analysis program. Following, the teratogenic index (TI) was determined, derived from LC_50_ and EC_50_ for malformations (ASTM, 2012). The weight, body lengths (TBL, SVL and TAL), daily body mass and TBL increment, were analysed through a Nested one-way analysis of variance (ANOVA) followed by Dunnett’s *post hoc* test to discriminate statistically significant differences between DMSO treatments and control (GraphPad Prism software version 8.0.2 (263), USA). The validity of the models (including normality and homoscedasticity) was checked by the evaluation of the residual plots.

The cell viability data (*in vitro* assays) was analysed to estimate lethal concentrations (LC_50_, LC_25_ and LC_10_), through a nonlinear regression fitting curve (four-parameter dose-response) using the GraphPad Prism software version 8.0.2 (263). Two-way analysis of variance ANOVA was conducted to analyse the cytotoxicity interaction between exposure time and concentration followed by Tukey’s *post hoc* test for comparison of means. The comparative assessment of the sensitivity of *in vivo* and *in vitro* assays was done through the ecotoxicological parameters that were calculated (LCs and ECs).

## 3 Results

### 3.1 Embryos teratogenicity assays

In the embryos assays, the survival rate of the controls*,* for *X. laevis* and *P. perezi*, was greater than 90% and malformations incidence was below 10%, meeting the validity criteria of the FETAX guideline (ASTM, 2012).

*Xenopus laevis* embryos exhibited a slightly higher sensitivity to DMSO than *P. perezi* embryos, as shown by the 96 h-LC_50_, which was 2.19% for the former species and 3.19% for the later (Table 1). The mortality of embryos of both species increased gradually with increasing concentrations of DMSO. *Xenopus laevis* embryos displayed 100% mortality at 2.97% and 4.01% treatments (Supplementary Figure SI1; Supplementary Table SI1), whereas for *P. perezi* 100% mortality was only observed from the treatment 4.01% onwards (Supplementary Figure SI1; Supplementary Table SI1). At 96 h, all *X. laevis* surviving organisms had hatched, whereas in the assay with *P. perezi*, the organisms from the 2.97% DMSO treatment (the highest concentration with surviving embryos) did not hatch, while at the lowest concentrations, 95%–100% hatched.

**TABLE 1:** Estimated median lethal concentrations (LC_50_), median effective concentrations for malformations (EC_50_) and teratogenic index (TI) of DMSO for embryos and tadpoles of *Xenopus laevis* and *Pelophylax perezi*, after 96 h of exposure. Values within parentheses represent the 95% confidence intervals.

Species	Assay (organism)	LC_50_ (% v/v)	EC_50_ (% v/v)	TI
*Xenopus laevis*	Embryos	2.19 (1.95–2.42)	1.44 (1.35–1.55)	1.52
	Tadpoles	2.56 (2.46–2.70)	_	_
*Pelophylax perezi*	Embryos	3.19 (2.83–3.55)	_	_
	Tadpoles	3.41 (3.04–3.78)	_	_

**FIGURE 1:**
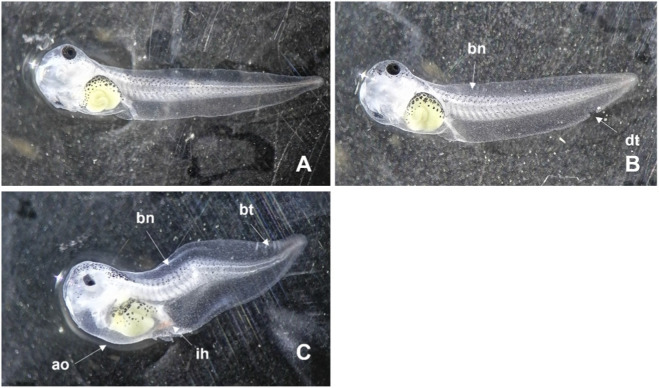
Images illustrating the malformations observed during the embryo teratogenicity assay with *Xenopus laevis*, after 96 h of exposure to DMSO. **(A)** Treatment Control; **(B)** Treatment 1.21% DMSO; **(C)** Treatment 2.20% DMSO. bn: bent notochord; dt: damaged tail; bt: bent tail; ao: abdominal oedema; ih: intestinal haemorrhages.

**FIGURE 2:**
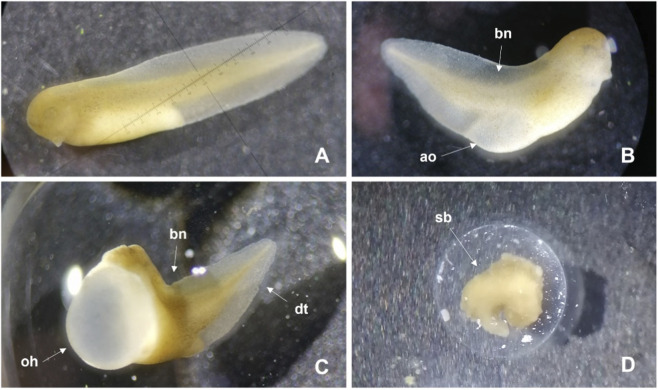
Images illustrating the malformations observed during the embryo teratogenicity assay with *Pelophylax perezi*, after 96 h of exposure to DMSO. **(A)** Treatment Control; **(B)** Treatment 1.63% DMSO; **(C)** Treatment 2.20% DMSO; **(D)** Treatment 2.97% DMSO. bn: bent notochord; ao: abdominal oedema; dt: damaged tail; oh: oedema in the heart; sb: stunted body.

After 96 h of exposure to DMSO, the embryos’ malformations were consistent in both species, including predominantly: bent notochord, damaged tail (ripped or with white stains), and oedema (e.g., abdominal and in the heart) (Figures 1, 2). In the case of *X. laevis*, the presence of intestinal haemorrhages was also frequent (Figure 1C). These malformations started to be detected, in *X. laevis*, at the lowest tested concentration (0.89%), in 7.9% of the organisms, with incidence increasing with increasing concentrations, reaching 100% of malformations in the organisms exposed to 2.20% DMSO (the highest concentration with surviving organisms). For *X. laevis*, the estimated 96 h-EC_50_ for malformations was 1.44% of DMSO and the respective TI was 1.52 (Table 1). In *P. perezi* assay, 72% of the organisms developed malformations in the lowest tested concentration (1.63% of DMSO) and 100% were affected in the following treatments (2.20% and 2.97%). Surviving organisms from the 2.97% treatment (highest concentration with survivors) exhibited completely stunted bodies (Figure 2D), impairing the measurement of their body lengths. For the two studied species, the organisms from the controls did not show visible malformations (Figures 1A, 2A).

**FIGURE 3:**
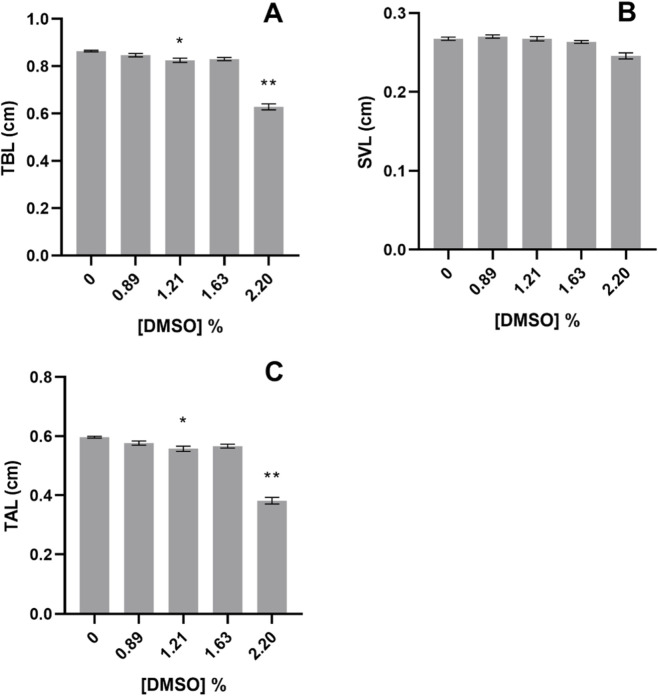
Body lengths of the *Xenopus laevis* after 96 h of exposure to DMSO, during the embryo teratogenicity assay. **(A)** Total body length, TBL; **(B)** Snout-to-vent length, SVL; **(C)** Tail length, TAL. All values are presented as mean ± SE. * and ** denote a statistically significant differences to control (0%) (Dunnett’s test; *p < 0.05* and *p < 0.001*, respectively).

The length of *X. laevis* organisms tended to decrease with increasing concentrations of DMSO (Figure 3). The TBL and TAL were significantly lower than control in organisms exposed to 1.21% and 2.20% DMSO (Nested Way ANOVA, F_4,12_ = 77.61 and F_4,12_ = 86.74; *post hoc* analysis, *p < 0.05*, Figures 3A,C). However, no significant alterations were recorded for SVL of organisms exposed to DMSO (Nested Way ANOVA, F_4,12_ = 0.80; *post hoc* analysis, *p = 0.55*, Figure 3B).

**FIGURE 4:**
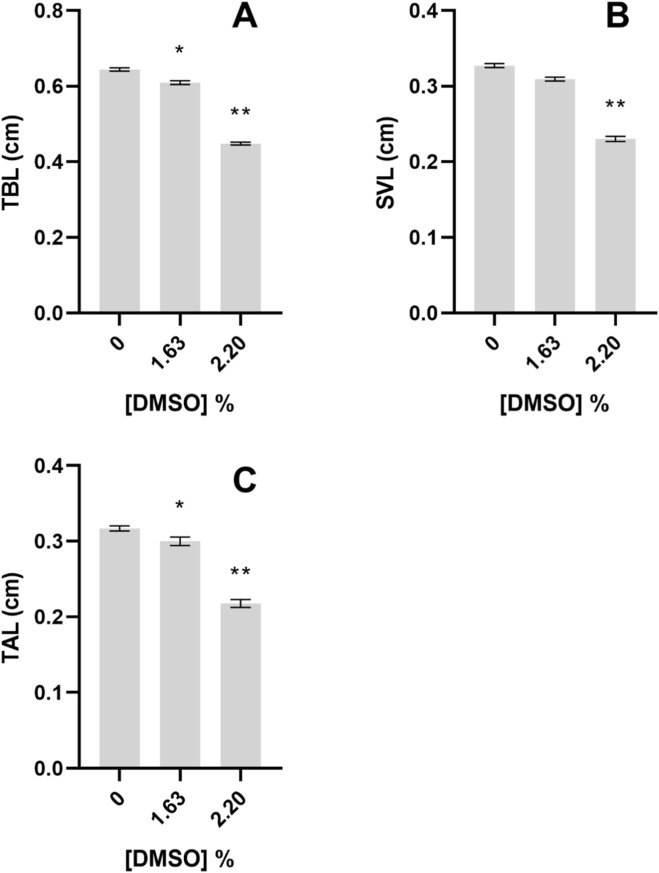
Body lengths of the *Pelophylax perezi* after 96 h exposure to DMSO, during the embryo teratogenicity assay. **(A)** Total body length, TBL; **(B)** Snout-to-vent length, SVL; **(C)** Tail length, TAL. All values are presented as mean ± SE. * and ** denote a statistically significant differences to control (0%) (Dunnett’s test; *p < 0.05* and *p < 0.001*, respectively).

In *P. perezi*, the same trend of decreasing body lengths was observed. The length of surviving organisms from the 2.97% treatment was not included in this analysis as the severe growth impairment (stunted body) prevented their measurement (Figure 4). Organisms exposed to 1.63% and 2.20% of DMSO presented significantly lower TBL (Nested Way ANOVA, F_2,6_ = 146.5; *post hoc* analysis, *p < 0.05*, Figure 4A) than those exposed to the control. *Pelophylax perezi* organisms exposed to 2.20% DMSO also exhibited significantly shorter SVL than controls (Nested Way ANOVA, F_2,6_ = 71.75; *post hoc* analysis, *p < 0.05*, Figure 4B), whereas TAL, as observed for TBL, decreased significantly in organisms from 1.63% to 2.20% DMSO treatments (Nested Way ANOVA, F_2,6_ = 146.5; *post hoc* analysis, *p < 0.05*, Figure 4C).

### 3.2 Acute toxicity assay with tadpoles

For both species, the percentage of dead tadpoles in the controls was below 10% and no tadpole showed malformations, meeting the validity criteria of the ASTM international guideline (ASTM, 2014).

As observed in the embryo’s assays, *X. laevis* tadpoles exhibited a slightly higher sensitivity to DMSO than those of *P. perezi*, as demonstrated by the estimated 96 h-LC_50s_ of 2.56% (*X. laevis)* and 3.41% (*P. perezi*) (Table 1). In the assay with *X. laevis,* 100% mortality was observed in the treatments 3.48% and 4.18% DMSO, with a single tadpole surviving at 2.90% DMSO (Supplementary Figure SI2). Considering *P. perezi*, 100% mortality was observed at 4.06% and 4.87% DMSO (Supplementary Figure SI2).

**FIGURE 5:**
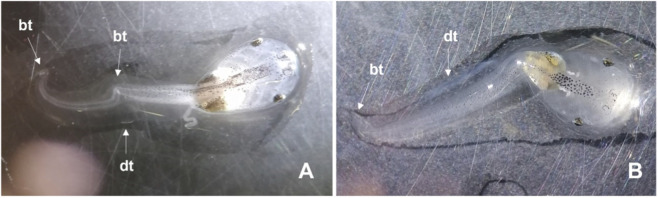
Malformations observed in *Xenopus laevis* tadpoles after 96 h exposure to DMSO. **(A)** Treatment 2.02% DMSO; **(B)** Treatment 2.42% DMSO. bt: bent tail; dt: damaged tail.

**FIGURE 6:**
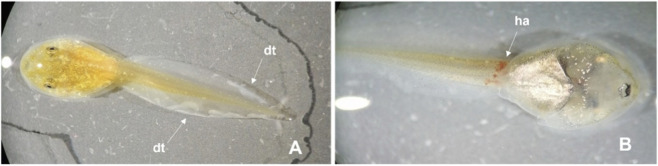
Malformations observed in *Pelophylax perezi* tadpoles after 96 h exposure to DMSO. **(A)** Treatment 2.35% DMSO; **(B)** Treatment 2.82% DMSO. dt: damaged tail; ha: haemorrhage.

In terms of malformations, *X. laevis* tadpoles from the lowest concentration of DMSO tested (1.40%), exhibited no visible malformations but from the next concentration (1.68%) on, all surviving tadpoles showed one or more malformations. In *P. perezi*, 87% of the tadpoles exposed to the lowest concentration tested (1.63% DMSO) exhibited malformations, 93% in the concentration 1.96%, and 100% tadpoles in the subsequent treatments. Overall, upon exposure to DMSO, the malformations observed in tadpoles of both species included damaged tails (ripped or with white stains) and internal haemorrhages (e.g., in the heart and intestines) (Figures 5, 6). Besides these two phenotypic effects, the presence of bent tail was observed in the *X. laevis* tadpoles (Figure 5).

**FIGURE 7:**
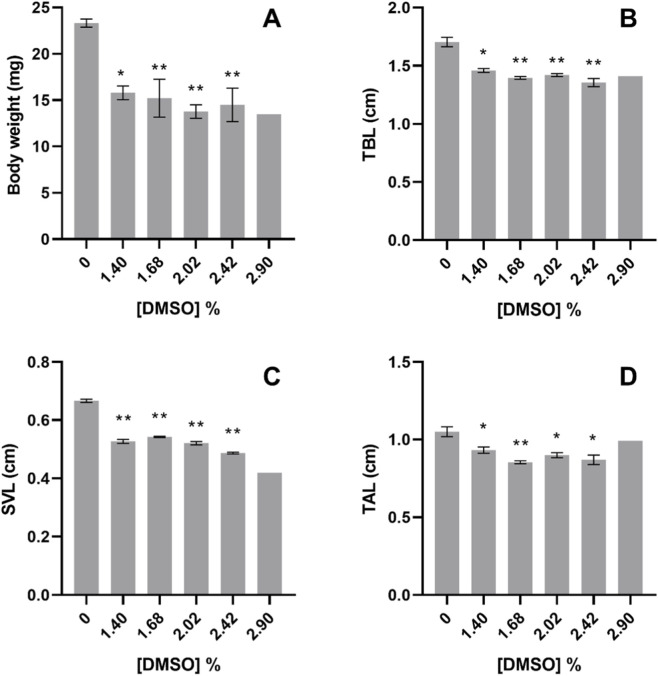
**(A)** Body weight, **(B)** Total body length - TBL, **(C)** Snout-to-vent length–SVL and **(D)** Tail length–TAL, of *Xenopus laevis* tadpoles after 96 h exposure to DMSO. In treatment 2.90%, n = 1. All values are presented as mean ± SE. * and ** denote a statistically significant differences to control (0%) (Dunnett’s test; *p < 0.05* and *p < 0.001*, respectively).

**FIGURE 8:**
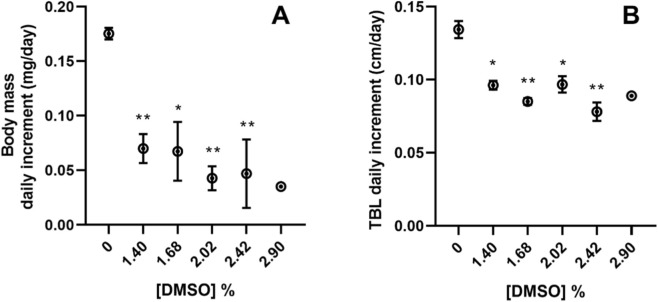
**(A)** Daily body mass increment and **(B)** Daily total body length (TBL) increment of *Xenopus laevis* tadpoles after 96 h of exposure to DMSO. In treatment 2.90%, n = 1. All values are presented as mean ± SE. * and ** denote a statistically significant differences to control (0%) (Dunnett’s test; *p < 0.05* and *p < 0.001*, respectively).

After 96 h of exposure, the body weight of *X. laevis* tadpoles decreased with increasing concentrations of DMSO. At 1.40, 1.68, 2.02% and 2.42% treatments, tadpoles were significantly lighter compared to the control (Nested Way ANOVA, F_5,20_ = 8.996; *post hoc* analysis, *p < 0.05*, Figure 7A). According to their weight, tadpoles’ body mass daily increment was significantly lower than control in organisms exposed to 1.40, 1.68, 2.02% and 2.42% (Nested 1-Way ANOVA, F_5,20_ = 9.939; *post hoc* analysis, *p < 0.05*, Figure 8A).

The TBL of the *X. laevis* tadpoles exposed to 1.40, 1.68, 2.02, 2.42% DMSO was also significantly lower than in control (Nested 1-Way ANOVA, F_5,20_ = 9.382; *post hoc* analysis, *p < 0.05*, Figure 7B), as well as, the TBL daily increment was significantly lower than in the tadpoles from the control (Nested 1-Way ANOVA, F_5,20_ = 8.195; *post hoc* analysis, *p < 0.05*, Figure 8B). The same significant decrease in the lengths of the exposed *X. laevis* tadpoles occurred for the SVL (Nested 1-Way ANOVA, F_5,20_ = 17.10; *post hoc* analysis, *p < 0.05*, Figure 7C), and the TAL (Nested 1-Way ANOVA, F_5,20_ = 5.225; *post hoc* analysis, *p < 0.05*, Figure 7D).

**FIGURE 9:**
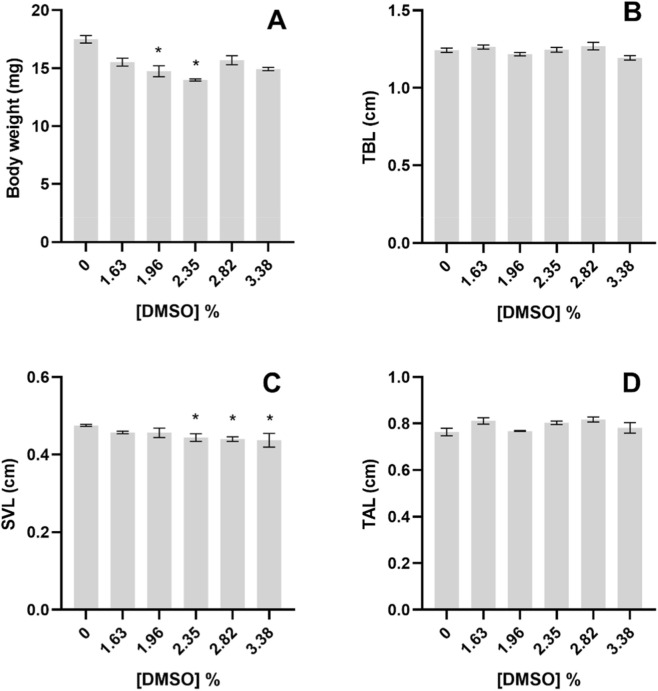
**(A)** Body weight, **(B)** Total body length - TBL, **(C)** Snout-to-vent length–SVL and **(D)** Tail length–TAL, of the *Pelophylax perezi* tadpoles after 96 h exposure to DMSO. All values are presented as mean ± SE. * denotes a statistically significant difference compared to control (0%) (Dunnett’s test; *p < 0.05*).

**FIGURE 10:**
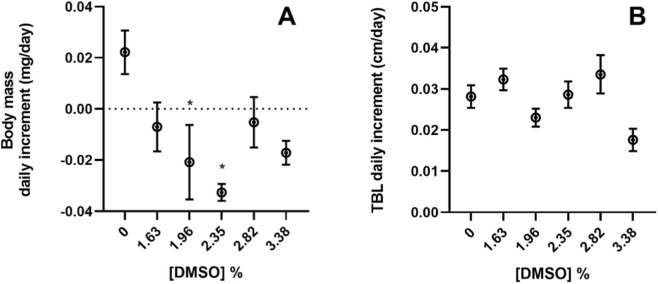
**(A)** Daily body mass increment and **(B)** Daily total body length (TBL) increment of the *Pelophylax perezi* tadpoles exposed to DMSO during 96 h. All values are presented as mean ± SE. * denotes a statistically significant difference compared to control (0%) (Dunnett’s test; *p < 0.05*).

For *P. perezi*, the body weight also tended to decrease in the tadpoles exposed to DMSO, with organisms being significantly lighter than control in 1.96% and 2.35% DMSO treatments (Nested 1-Way ANOVA, F_5,23_ = 4.024; *post hoc* analysis, *p < 0.05*, Figure 9A). Tadpoles’ body mass daily increment was significantly lower in the same treatments (Nested 1-Way ANOVA, F_5,23_ = 3.813; *post hoc* analysis, *p < 0.05*; Figure 10A).

Regarding body lengths (Figures 9B–D), no significant effects of DMSO in *P. perezi* tadpoles were found, except in terms of SVL which was significantly smaller than control in organisms exposed to 2.35, 2.82% and 3.38% of DMSO (Nested 1-Way ANOVA, F_5,69_ = 2.612; *post hoc* analysis, *p < 0.05*, Figure 9C). Accordingly, the TBL daily increment was also not significantly affected by DMSO exposure in *P. perezi* tadpoles (Figure 10B).

### 3.3 Cytotoxicity assays with A6 and XTC-2 cell lines of *Xenopus laevis*

**FIGURE 11:**
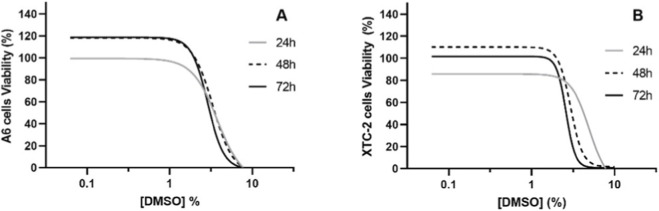
Viability of A6 **(A)** and XTC-2 **(B)** cells exposed to DMSO during 24, 48 and 72 h. Fitted curve (4P) to the viability data is presented and viabilities are calculated as percentage of the control.

Analysing Figure 11, which represents the fitted curves of the nonlinear regression of A6 and XTC-2 viabilities, it is possible to verify that cells’ viability decreases with increasing concentrations of DMSO. Cellular viability decrease starts to accentuate from the treatment 2% of DMSO treatment onwards and approaches zero from 6% onwards (Figure 11). The DMSO lethal concentrations (LC_50_, LC_25_, and LC_10_) for the tested cell lines, extrapolated from the curves and depicted in Table 2, corroborate these findings. The estimated DMSO LC_c_ for A6 and XTC-2 cells were similar in each timepoint (24, 48, 72 h) (Table 2). Specifically, the 72 h-LC_50_ for A6 and XTC-2 cell lines were, respectively, 3.10% and 2.62% (Table 2). The confidence intervals of the calculated LC_c_, values of the cell lines overlap, suggesting a similar sensitivity to DMSO. In the case of A6, the concentration of DMSO and the exposure time significantly affected the viability of the cells (Two-way ANOVA F_9,80_ = 81.47, *p < 0.0001* and F_2,80_ = 4.07, *p < 0.05*, respectively). However, no significant interaction between both factors was found. Comparing the exposure periods, significant differences were found in the viability of cells at 24 h and 48 h (Tukey’s multiple comparisons test, *p < 0.05*). The LC_50_ values remained similar across exposure periods, 3.50, 3.55, and 3.10%, respectively, for 24, 48, and 72 h, with confidence intervals overlapping (Table 2). For XTC-2, the exposure time did not significantly affect the cells’ viability (Two-way ANOVA F_2,80_ = 2.36, *p = 0.10*). However, the concentration of DMSO significantly affected the viability of the cells (Two-way ANOVA F_9,80_ = 60.92, *p < 0.0001*) with an exposure time dependency, since a significant interaction between both factors was observed (Two-way ANOVA F_18,80_ = 1.88, *p < 0.05*).

**TABLE 2:** Estimated concentrations of DMSO causing 50%, 25% and 10% lethality (LC_50_, LC_25_ and LC_10_, respectively) on A6 and XTC-2 cell lines, after 24, 48 and 72 h of exposure. Values within parentheses represent 95% confidence intervals.

Exposure period (h)	Toxicity	A6 cells	XTC-2 cells
24	LC_50_ (% v/v)	3.50 (2.72–4.26)	4.28 (3.51–4.91)
	LC_25_ (% v/v)	2.43 (1.69–3.16)	2.90 (2.04–3.79)
	LC_10_ (% v/v)	1.64 (0.81–2.58)	_
48	LC_50_ (% v/v)	3.55 (2.74–4.41)	3.06 (2.31–3.83)
	LC_25_ (% v/v)	2.80 (2.05–3.52)	2.60 (1.99–3.38)
	LC_10_ (% v/v)	2.37 (1.67–3.11)	2.29 (1.72–3.15)
72	LC_50_ (% v/v)	3.10 (2.86–3.35)	2.62 (2.17–3.55)
	LC_25_ (% v/v)	2.55 (2.35–2.77)	2.28 (1.93–3.27)
	LC_10_ (% v/v)	2.23 (2.02–2.45)	2.00 (1.56–3.12)

## 4 Discussion

Lethality induced by DMSO was similar in the early life stages of both tested species, *X. laevis* and *P. perezi*, with the latter species being slightly more resistant, at embryos and tadpoles’ stages. The sensitivity of early life stages of *X. laevis* to chemical exposure, relative to other amphibian species, has been shown to be chemical-dependent. Some studies report *X. laevis* as more sensitive, while others describe it as more resistant to various xenobiotics (e.g., Adams et al., 2021; Frątczak et al., 2025). The ecotoxicity data, available in the literature, that allows the comparison of the sensitivity of two model species used in the present work is limited. As a rare example, Santos et al. (2023a); Santos et al. (2023b) assessed the toxicity of wildfire ashes to tadpoles of *X. laevis* and *P. perezi* and found the latter species to be more resistant, being in line with the results here obtained. The observed higher resistance of *P. perezi* embryos and tadpoles could be related to the fact that the tested organisms were obtained from a field population. In the field, organisms are usually exposed to some environmental perturbations (absent in laboratory cultures) that may induce the activation of physiological mechanisms related, for example, to detoxification processes, enabling the organisms to respond faster to the exposure to DMSO. Moreover, the epigenetic impacts stemming from exposure to subtle natural perturbations may endure, potentially leading to the activation of some of those mechanisms that enable the organisms to cope with environmental stress (Verheijen et al., 2019). Consequently, this phenomenon could enhance the resilience of natural populations, fostering a heightened resistance to chemical contamination.

When comparing life stages, the obtained LC_50_ values for DMSO suggest a slightly higher sensitivity of embryos relatively to tadpoles in the two studied species. The ability of other common solvents, such as methanol and methylene chloride, to induce higher mortality rates in amphibian embryos than in tadpoles has been shown in *Rana temporaria* (Marquis et al., 2006). These authors, who assessed the toxicity of DMSO to embryos of *R. temporaria*, with and without the jelly coat removed, reported higher toxicity of DMSO in organisms where the jelly coat was not removed. The explanation proposed by Marquis et al. (2006) to justify such paradoxical results was that some components present in the jelly coat could react with DMSO, turning it more toxic to the embryos. This hypothesis could also explain the higher sensitivity of embryos relative to tadpoles. Furthermore, tadpoles by having the liver developed (NF 47) and additional mechanisms of detoxification activated, could be capable of metabolizing and excreting DMSO, leading to a lower toxicity (Zorn and Mason, 2001).

The 96 h‐LC_50_ determined for *X. laevis* embryos (2.19%; 95% CL of: 1.95–2.42) was slightly higher than the pooled 96 h‐LC_50_ of 1.92% (two trials) previously estimated by Dresser et al. (1992), also following the FETAX guideline. Despite the slight difference between both works, since Dresser et al. (1992) computed large 95% confidence interval (1.73%–18.88%), which comprises the LC_50_ estimated in the present work, the LC_50_ values can be considered similar. Furthermore, the marginal observed difference may be associated with the absence of the jelly coat on the tested *X. laevis* embryos, an approach the authors adopted, aiming to enhance the susceptibility of the embryos to teratogenicity (Dresser et al., 1992). Considering the environmental relevance of the data, in the present study, the jelly coat, which plays a crucial role in the fertilisation process and development of amphibian’s eggs (Turani et al., 2020), was not removed. Other authors suggested that the jelly coat works as a protective layer and may reduce the toxicity of environmental agents in embryonic stages (Aronzon et al., 2011). However, as mentioned above, an opposite finding was described by Marquis et al. (2006), who reported a higher sensitivity to DMSO of embryos with jelly coat than without. Nonetheless, *R. temporaria* embryos were more affected than those of *X. laevis* and *P. perezi*, exhibiting a mortality rate of 87.5% ± 5.2% at 0.01% of DMSO (limit concentration recommended for solvents; OECD, 2019), after 96 h exposure. According to the present results and the literature, the sensitivity of amphibians to solvents varies among species, with 96 h‐LC_50_ values ranging from <0.01% (*R. temporaria* embryos) to 3.19% and 3.41% (*P. perezi* embryos and tadpoles, respectively). Furthermore, the survival of *Lithobates pipiens* tadpoles was not affected after 96 h of exposure to concentrations of DMSO up to 0.01% (Young et al., 2020), demonstrating, as the present work, that concentrations of DMSO recommended by the guideline (≤0.01%) do not affect the survival of the exposed amphibians. However, in some species and depending on life stage and solvent used this concentration may be too high and may induce adverse effects.

Besides the LC_50s_ described for *X. laevis* and *P. perezi* early life stages, the ones estimated for other aquatic model species were also much higher than the OECD recommendation, such as for the microcrustacean *Daphnia magna* (48 h-LC_50_: 1.17%, Andrade-Vieira et al., 2022, and 48 h-LC_50_: 0.50%; Huang et al., 2018, both for immobilization), the crustaceans *Artemia franciscana and Allorchestes compressa* (48 h-LC_50_: 5.64% and 5.41%, *respect*ively; (Huang et al., 2018) and the microalgae *Raphidocelis subcapitata* (72-h EC_50_: 2.14%, for growth inhibition; Andrade-Vieira et al., 2022), explaining why DMSO is frequently used as a solvent in much higher concentrations (e.g., Boyd et al., 2021; de Paula et al., 2022; Hoyberghs et al., 2021; Thorel et al., 2020).

DMSO exposure had, in general, a limited impact on hatching. After 96 h of exposure, all *X. laevis* hatched as well as the majority of *P. perezi*, except in the highest concentration with surviving organisms (2.97% DMSO), where organisms development was stunted and none of the survivors hatched. As well, exposure to DMSO caused malformations in many of the exposed organisms (embryos and tadpoles of both species), and although the tested concentrations were much higher than the maximum recommended to be used in lethal ecotoxicity assays (in order to calculate the LC_50s_), its ability to induce morphological deformities to amphibians was previously demonstrated at much lower concentrations. For instance, tadpoles of *L. pipiens* acutely exposed to DMSO showed significantly reduced interorbital distance at 0.0025%–0.01%, however, exposures to low concentrations (≤0.001%) increased this morphometric parameter, with the authors explaining, as a possible cause, the fact that randomly smaller tadpoles were assigned to control (Young et al., 2020). The most common malformations detected in the embryos’ assays of both species were bent notochord, damaged tail, and oedema, and in tadpoles tail and internal haemorrhages. Dresser et al. (1992), also described the induction of skeletal abnormalities and abdominal oedemas in embryos of *X. laevis* exposed to >1% DMSO, the presence of loose or displaced gut coiling caused by oedema, which was also observed in some of the embryos of *X. laevis* exposed to DMSO, in this study. The development of abdominal oedemas might compromise the normal functioning of the digestive system, as the nutritional intake is influenced by the length and configuration of the digestive tract (Bloom et al., 2013). DMSO also induced the development of oedemas in embryos of other aquatic organisms. Embryos of *D. rerio* exposed for 48 h to 3.2% DMSO developed pericardial oedemas (Turner et al., 2012), which were also found in organisms exposed to DMSO in the present study.

Damaged tail was very frequent in *X. laevis* and *P. perezi,* both in embryos and tadpoles. It consisted of white stains on the skin or ripped skin suggesting an ability of DMSO to induce cellular death in these areas. Indeed, Orzechowski et al. (2001) suggested that DMSO negatively regulates myogenesis accelerating muscle cell apoptosis. Furthermore, this phenomenon was previously described in embryos of *D. rerio* exposed to 2% of DMSO, which, besides the presence of oedemas, also displayed abnormalities in the tissue named as tissue deviation from the tail or/and the body, and described as cell death (Hoyberghs et al., 2021). In the present study, the effects of DMSO were especially severe at 2.97%, in the assay with embryos of *P. perezi*, where all surviving embryos developed with completely stunted bodies, which may compromise their health and survival by impairing different physiological systems. Bearing in mind that effects during early life stages might result in adverse effects on later stages, alterations on skeletal structure and tail deformations might impair swimming behaviour of tadpoles. Changes on behaviour patterns could impair food foraging, mating and avoiding predators, which might have consequences at individual’s fitness and subsequently translate to the population level (Amiard-Triquet, 2009; Weis et al., 2001).

The data obtained in this study only allowed the estimation of the DMSO teratogenic index (TI) for the embryos of *X. laevis* (TI = 1.52), as the EC_50_ for malformations could only be computed in this assay due to the presence of malformations in 100% of the organisms in most of the treatments with DMSO in the other three assays. According to ASTM (2012), these results indicate that DMSO has strong teratogenic potential for *X. laevis* embryos (TI ≥1.5). This value was higher than the TI values reported by Dresser et al. (1992) for *X. laevis* embryos (two replicate trials), which were 1.20 and 1.24 indicating low teratogenicity of DMSO. Although in the present study, the TI could not be estimated for the other assays, most of the organisms exposed to DMSO displayed one or more malformations resulting from DMSO exposure, demonstrating the teratogenic potential of DMSO. Regarding this, malformations at early life stages, namely, on development, might compromise later stages fitness and their long-term survival (Amiard-Triquet, 2009; Weis et al., 2001). In addition, the accumulation of developmental malformations can ultimately lead to the death of the organism.

Biometric parameters of both species and life stages were affected by DMSO exposure, with the embryos of *P. perezi* and the tadpoles of *X. laevis* being the most affected. In *P. perezi*, DMSO exposure led to reductions in several length parameters, resulting in smaller organisms. DMSO also decreased the length (both life stages) and weight (tadpoles) of *X. laevis,* suggesting an adverse effect on the growth of this species, which was more notorious on tadpoles’ assay where the daily increment of body mass and length were also lower. These results are in accordance with those found by Dresser et al. (1992), whom also reported a reduction in the length of *X. laevis* larvae exposed to DMSO, at concentrations >1%.

Results showed that DMSO retarded the growth of *X. laevis* embryos and tadpoles and *P. perezi* embryos. In such a crucial life stage of amphibians, the smaller size of the organisms might lead to a prolonged period in the tadpole stage, once the body size is one of the main quantitative factors that regulate the onset of metamorphosis (Adolph, 1931). Moreover, it is suggested that, in anurans, the size at metamorphosis may affect the juvenile physiology, performance, and survival, and later, adult reproduction (Bekhet et al., 2014; Cabrera-Guzmán et al., 2013). Body weight also constitutes a decisive factor in metamorphosis (Adolph, 1931), and it was also affected in *X. laevis* and *P. perezi* tadpoles by exposure to DMSO. Their body weights decreased with increasing concentrations of DMSO, which may be associated with an impact on feeding behaviour, since food was added to the assay, or with the absorption of the vitelline reserves to cope with the energetic expenditure required for the activation of mechanisms of detoxification. This weight loss was confirmed by the much lower daily body mass increments in the exposed tadpoles than in the controls. Besides delaying the organisms’ development, reduced body size and mass can affect the viability of the larvae or the metamorphs in many ways, such as increasing their vulnerability for mortality sources, predation, ecological competition, and starvation (Cabrera-Guzmán et al., 2013).

Overall, data suggest that in *in vivo* assays with early life stages of anuran, the recommended concentration of DMSO (0.01%) should be respected, especially when evaluating apical endpoints such as malformations, biometric parameters, and behaviour. However, this solvent concentration can be slightly higher if a highly hydrophobic compound needs to be dissolved, and the mortality is the only endpoint assessed for a first screening characterization of lethal toxicity. It is also important to keep in mind that the sensitivity of the organisms to DMSO might be different between species and life stages, especially while using *X. laevis* as a model in toxicology. Besides, even at low concentrations, DMSO can induce masked harmful effects in long-term exposures (Turner et al., 2012) and synergetic effects with other chemicals (Kim and Lee, 2021).

Regarding the *in vitro* assays, the estimated LCs for both cell lines (A6 and XTC-2) were similar in the three timepoints, and a decrease in cells’ viability started to accentuate from concentration 2% onwards. Other studies reported similar toxicity of DMSO in different cells and organisms. A decrease in the viability of rat retinal ganglion cells (RGC-5 cell line) was observed in concentrations higher than 1%, and the LC_50_ estimated after 24 h of exposure was 2.14% (Galvao et al., 2014), slightly lower than the ones estimated in the present study for A6 and XTC-2 cells (3.50% and 4.28%, respectively). The viability of human lymphocytes was not affected up to concentrations of 2.5% DMSO. However, at 1% and 2% their relative proliferation index was reduced (Costa et al., 2017). Several studies have highlighted the importance of previously overlooked effects of DMSO. The work from Verheijen et al. (2019) demonstrated that exposure of human hepatic and cardiac microtissues to 0.1% DMSO, deemed a safe concentration, led to differently expression of over 2000 genes involved in several pathways related with metabolism, namely, citric acid cycle, respiratory electron transport and glucose metabolism, pathways associated with vesicle-mediated transport and with cellular responses to stress, such as reactive oxygen species and cellular ATP production. Other authors described non-lethal alterations in other *in vitro* models, namely, mitochondrial alterations at 1% DMSO in astrocytes (Yuan et al., 2014), decreased viability at 0.5% DMSO, and increased Parp-1 and caspase-3 cleavage at 5% DMSO in human fibroblast-like synoviocytes, indicating early signs of cellular death (Gallardo-Villagrán et al., 2022). These results confirm that although being widely used in many therapeutic applications, in cryopreservation, and as a solvent, DMSO has toxic effects in concentrations generally accepted as low toxic *in vitro* and confirms that, as previously stated, its use and applicable concentrations should be reconsidered (Galvao et al., 2014).

The 72-h LC_50_ values of DMSO for the A6 and XTC-2 cell lines were within less than one order of magnitude of the 96-h LC_50_ values observed in *X. laevis* and *P. perezi* embryos and tadpoles, with notable overlap among the 95% confidence intervals (e.g., between XTC-2 cells and *X. laevis* embryos). *In vitro* assays with amphibian cell lines and *in vivo* assays conducted on early developmental stages yielded complementary and relevant ecotoxicological information. As expected, given the nature of the endpoints evaluated, the early life stages provided broader organism-level responses and appeared slightly more sensitive overall. Importantly, the comparison between the two systems also warrants consideration of the cellular origin used *in vitro*; for instance, XTC-2 cells, derived from early developmental stages of *X. laevis*, provide a biologically meaningful analogue for comparison with tadpoles. As such, these findings suggest that *in vitro* assays using amphibian cell lines could be effectively employed as preliminary screening tools in ecotoxicity assessments. This strategy has the potential to reduce the number of animals to be used in subsequent *in vivo* testing phases by providing information for the refinement of concentrations to be tested in *in vivo* assays, thereby supporting both reduction and replacement. Thus, incorporating *in vitro* assays as initial screening steps may substantially contribute to decrease animal usage in the early tiers of ecological risk assessment for chemicals, thereby aligning with the 3Rs principles of animal experimentation. To further validate this approach, additional studies involving diverse chemicals and cell lines—such as amphibian skin cell lines representing critical exposure pathways—are warranted to more accurately evaluate the suitability of *in vitro* methods as alternatives to *in vivo* assays. However, it has to be highlighted that lower concentrations of DMSO induced sublethal effects in the *in vivo* assays, which highlights its sensitive nature and applicability for xenobiotics’ hazard assessment, being considered good non-animal new approach methodologies to be used as *in vivo* assays at early tiers of ecological risk assessment for chemicals. Within the framework of the European Union Roadmap towards Phasing Out Animal Testing for Chemical Safety Assessments and the New Approach Methodologies (NAMs) development strategy, the integration of such cell-based systems aligns with the current policy direction to both replace and reduce the use of live organisms in ecotoxicological evaluations. Early life-stage assays remain recognized as valuable Replacement tools under Directive 2010/63/EU, since they involve non-independent feeding larvae; however, the EU also promotes a progressive reliance on NAMs, including cell-based bioassays, *in silico* models, and mechanistic testing approaches that enable preliminary hazard screening while minimizing *in vivo* testing. Thus, the combined and sequential use of cell line-based and early life-stage assays exemplifies the scientific and regulatory transition toward an integrated, tiered testing paradigm that supports the 3Rs and advances predictive environmental safety assessment.

## 5 Conclusion

Overall, obtained results suggest that DMSO sensitivity is generally comparable between *X. laevis* and *P. perezi*, and across developmental stages, despite the slightly lower LC_50_ values in embryos and *X. laevis*. While lethal and sublethal toxicity only occurred above 0.01%, the maximum concentration used in standard assays, it is advisable to keep DMSO concentrations as low as possible; since, induced malformations may affect later developmental stages and impact the fitness of the organisms, with potential consequences at the community and population levels.

Both *in vitro* and *in vivo* assays provided complementary insights into DMSO toxicity. Early life stages were slightly more sensitive, but comparable LC_50_ values indicate that *in vitro* assays, particularly those with XTC-2 cells, can effectively serve as surrogates for *in vivo* testing as it regards first screenings of ecotoxicity of organic solvents. At the same time, the results obtained with early life stages of amphibians highlight their value as ecologically relevant models to assess potential impacts of chemicals in wildlife, and must be considered at early phases of the risk assessment.

Further validation of *in vitro* approaches requires studies using additional cell lines relevant to amphibian exposure pathways (e.g., skin cells) and chemicals with diverse properties and modes of action to strengthen correlations with *in vivo* results and assess their suitability as alternative methods.

## Data Availability

The raw data supporting the conclusions of this article will be made available by the authors, on reasonable request and without undue reservation.
