# Supplementary material for: Comparative assessment of solvents toxicity using early life stages of amphibians and cell lines: a case study with dimethyl sulfoxide
Source: Front Toxicol. 2026 Jan 5;7:1672301. doi: 10.3389/ftox.2025.1672301 (PMC12812705; doi:10.3389/ftox.2025.1672301)
Supplement: Supplementary file 1 [file DataSheet1.docx]

**Supplementary information**


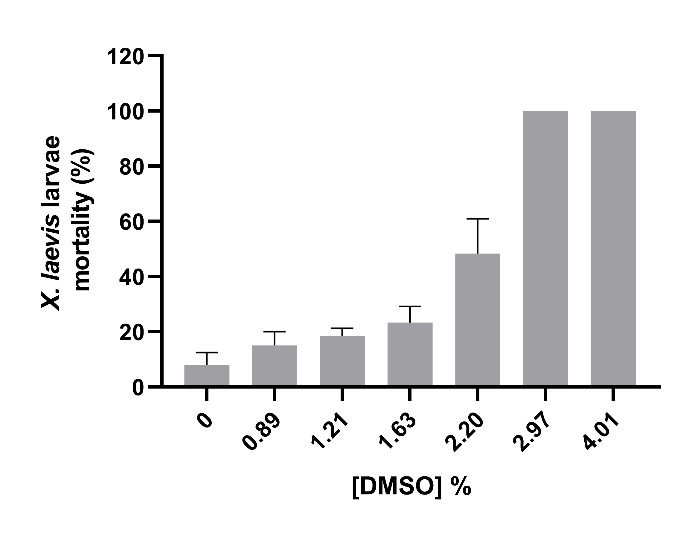
**
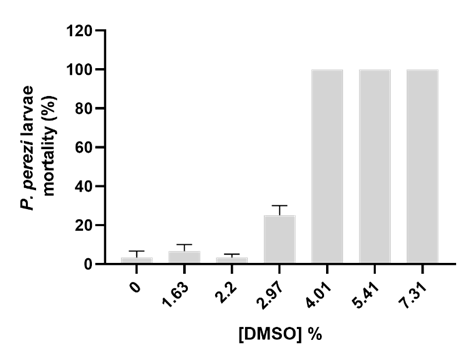
**

**Figure SI1.** Mortality of *Xenopus laevis* (left) and *Pelophylax perezi* (right) larvae (embryos assay) after 96 h exposure to different concentrations of DMSO. All values are presented as mean ± SE.


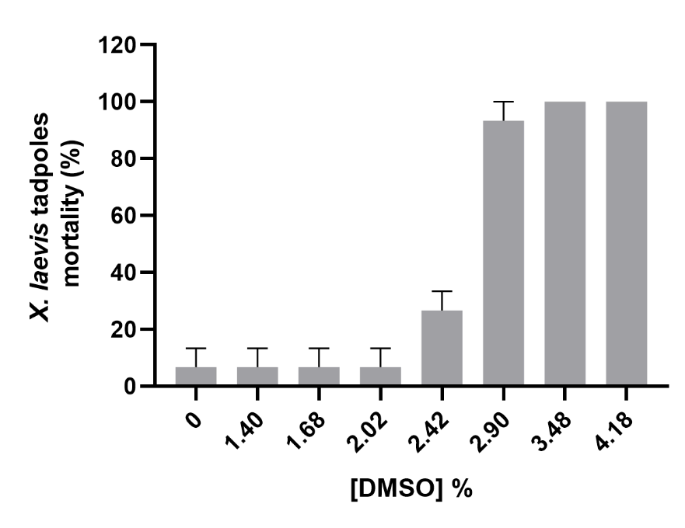

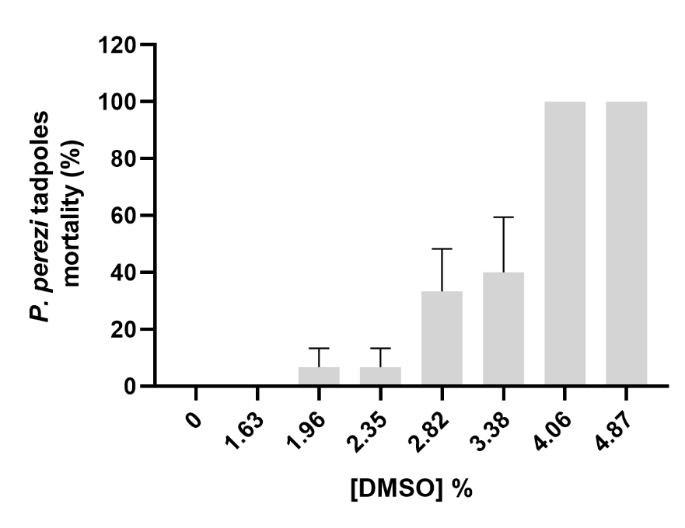


**Figure SI2.** Mortality of *Xenopus laevis* (left) and *Pelophylax perezi* (right) tadpoles (tadpoles assay) after 96 h exposure to different concentrations of DMSO. All values are presented as mean ± SE.

**Table SI1.** Number of dead organisms in the embryo and tadpoles’ assays performed with *Xenopus laevis* and *Pelophylax perezi*.

|  | **Treatments** | **Total number of organisms in the assay** | **Dead organisms at the end of the assay (96 h)** |
| --- | --- | --- | --- |
| *Xenopus laevis* | | | |
| **Embryo**  **assay** | Control (Fetax medium) | 60 | 8 |
|  | 0.89% DMSO | 60 | 9 |
|  | 1.21% DMSO | 60 | 11 |
|  | 1.63% DMSO | 60 | 14 |
|  | 2.20% DMSO | 60 | 29 |
|  | 2.97% DMSO | 60 | 60 |
|  | 4.01% DMSO | 60 | 60 |
| **Tadpoles assay** | Control (Fetax medium) | 15 | 1 |
|  | 1.40% DMSO | 15 | 1 |
|  | 1.68% DMSO | 15 | 1 |
|  | 2.02% DMSO | 15 | 1 |
|  | 2.42% DMSO | 15 | 4 |
|  | 2.90% DMSO | 15 | 14 |
|  | 3.48% DMSO | 15 | 15 |
|  | 4.18% DMSO | 15 | 15 |
| *Pelophylax perezi* | | | |
| **Embryo**  **assay** | Control (Fetax medium) | 60 | 2 |
|  | 1.63 | 60 | 4 |
|  | 2.20 | 60 | 2 |
|  | 2.97 | 60 | 15 |
|  | 4.01 | 60 | 60 |
|  | 5.41 | 60 | 60 |
|  | 7.31 | 60 | 60 |
| **Tadpoles assay** | Control (Fetax medium) | 15 | 0 |
|  | 1.63 | 15 | 0 |
|  | 1.96 | 15 | 1 |
|  | 2.35 | 15 | 1 |
|  | 2.82 | 15 | 5 |
|  | 3.38 | 15 | 6 |
|  | 4.06 | 15 | 15 |
|  | 4.87 | 15 | 15 |
